# Small Sample Coherent DOA Estimation Method Based on S2S Neural Network Meta Reinforcement Learning

**DOI:** 10.3390/s23031546

**Published:** 2023-01-31

**Authors:** Zihan Wu, Jun Wang

**Affiliations:** 1 School of Electronics and Information Engineering, Harbin Institute of Technology, Harbin 150001, China; 2 School of Information Science and Engineering, Harbin Institute of Technology (Weihai), Weihai 264200, China

**Keywords:** coherent DOA, small sample, meta−reinforcement learning (MRL), S2S neural network, Markov decision process (MDP)

## Abstract

Aiming at the existing Direction of Arrival (DOA) methods based on neural network, a large number of samples are required to achieve signal-scene adaptation and accurate angle estimation. In the coherent signal environment, the problems of a larger amount of training sample data are required. In this paper, the DOA of coherent signal is converted into the DOA parameter estimation of the angle interval of incident signal. The accurate estimation of coherent DOA under the condition of small samples based on meta−reinforcement learning (MRL) is realized. The meta−reinforcement learning method in this paper models the process of angle interval estimation of coherent signals as a Markov decision process. In the inner loop layer, the sequence to sequence (S2S) neural network is used to express the angular interval feature sequence of the incident signal DOA. The strategy learning of the existence of angle interval under small samples is realized through making full use of the context relevance of spatial spectral sequence through S2S neural network. Thus, according to the optimal strategy, the output sequence is sequentially determined to give the angle interval of the incident signal. Finally, DOA is obtained through one-dimensional spectral peak search according to the angle interval obtained. The experiment shows that the meta−reinforcement learning algorithm based on S2S neural network can quickly converge to the optimal state by only updating the gradient of S2S neural network parameters with a small sample set when a new signal environment appears.

## 1. Introduction

Direction of arrival (DOA) serves as an important research field. In particular, the algorithm represented by Multiple Signal Classification (MUSIC) [1,2,3,4] and Estimation of Signal Parameters using Rotational Invariance Techniques (ESPRIT) [5] has broken through the “Rayleigh limit” and achieved real super-resolution. In addition, the research on DOA of coherent signal sources in array direction finding is also a hot spot of spatial spectrum estimation technology. Therefore, traditional model-driven algorithms such as Spatial Smoothing Algorithm (SSMUSIC) [6,7,8,9] are widely used in the field of coherent DOA. While in the low-elevation altimetry problem of meter–wave radar, the direct signal and multipath signal belong to the spatially adjacent coherent source, and the classical super-resolution algorithm has limited resolution. The existing super-resolution algorithms such as MUSIC, ESPRIT, and ML can partially solve the DOA estimation problem under multipath conditions. However, the basis for ensuring good algorithm performance is that the actual received signal model meets the ideal plane wave model. Once the model is mismatched, the algorithm performance will decline sharply. This is the disadvantage of the existing physically-driven algorithms. Model-driven methods in practical engineering applications are always faced with severe challenges such as array error, low signal-to-noise ratio, and weak adaptability to complex environments.

Therefore, scholars have proposed various data-driven methods such as neural network and support vector machine (SVM) [10,11,12,13] in order to solve this problem. Because of their nonlinear characteristics, adaptive learning ability, and generalization ability, they have been applied to the field of array signal processing. Therefore, the nonlinear relationship between array output and signal direction can be learned to achieve direction finding. Therefore, the learning mode can be divided into unsupervised and supervised learning based on the current neural network DOA method. The neural network architecture can be divided into deep neural network (DNN), convolution neural network (CNN), deep convolution neural network (DCNN), convolutional recurrent neural network (CRNN), fully connected neural network (FC-NN), and other network architectures. The signal problems to be solved can be divided into incoherent signal, phase enhancement, and coherent signal.

Among them, a new unsupervised DNN neural network learning strategy for incoherent DOA is proposed in document [14], which can improve the degree of freedom of the array while maintaining a certain accuracy. In document [15], a DNN network framework composed of a multitask automatic encoder and a series of parallel multi-layer classifiers is proposed. The subsequent simulation results show that this method can adapt well to various array defects. When the defect is obvious, it can obtain the incoherent DOA with higher accuracy than the most widely studied MUSIC parameter method. In literature [16], a DCNN-based network architecture is proposed to learn the inverse transform from array output to DOA spectrum, so that incoherent DOA estimation can be effectively obtained in near real time. In addition, compared with the existing methods based on deep learning, the performance of DOA estimation is improved by using sparse prior. The simulation results also show that the method has advantages in the accuracy and computational efficiency of incoherent DOA. In literature[17], three neural network models of DNN, one-dimensional CNN, and two-dimensional CNN, and their optimization methods are proposed to reduce the phase distortion caused by multipath signals and enhance the phase characteristics of direct signals. The simulation results show that the proposed feature-to-feature learning method has superior DOA performance compared with the latest methods including physically driven methods and existing data-driven methods. In literature [18], a low-angle estimation phase enhancement method based on supervised DNN is proposed to reduce phase distortion and improve the accuracy of incoherent DOA estimation. Experimental results and real data results verify the effectiveness and feasibility of this method. However, at present, the number of literatures on neural network incoherent DOA increases in positive proportion to the number of researchers as the year progresses, but the literature on coherent DOA estimation is still too limited. Among them, a spatial filter and alternating multi-label classifier based on CRNN unit design are proposed in literature [19], which can recover the model of the arrival angle of coherent signals, and the model can still achieve high estimation accuracy with the help of Toeplitz matrix reconstruction, even if the number of sources is unknown. Finally, the simulation on the linear array shows that this method has great advantages over the latest FC-NN and traditional SS-MUSIC algorithm. In literature [20], depth learning is applied to estimate the direction of arrival of multiple narrowband signals with uniform linear array in coherent environment. First, a classification network based on logarithmic eigenvalues (LogECNet) is introduced to improve the detection accuracy of signal number in challenging scenarios, such as low signal-to-noise ratio and limited number of snapshots. Next, a multi-label classification model called Root Spectrum network (RSNet) is designed to estimate DOA using the number of signals inferred by LogECNet. Simulation results show that the proposed method not only improves the performance of signal number detection and angle estimation, but also reduces the complexity compared with previous schemes. In literature [21], a new hybrid model-based (MB)/data-driven (DD) DOA structure based on the classical MUSIC algorithm is proposed. This method enhances the important aspects of the original MUSIC structure through a specially designed neural structure, thus overcoming some limitations of the pure MB method, such as the inability to successfully locate coherent sources. The depth-enhanced MUSIC algorithm has higher resolution than the unchanged version. In document [22], a multi-objective joint learning (MOJL) model was proposed to mine the potential joint characteristics of multiple coherent signals, separate them into different subnets, reconstruct the data of each signal, and perform super-resolution DOA according to the output of the subnets. The simulation results show that the proposed method can separate the data of multiple coherent signals with a small error. The statistical results show that the proposed method is superior to the traditional physical-driven method and advanced data-driven method in terms of DOA accuracy, SNR, and array incompleteness generalization. However, these algorithms are only applicable to coherent or incoherent signals outside a beamwidth, and cannot solve the problem of DOA of spatially adjacent coherent sources within a beamwidth. Therefore, in document [23], the author proposes two separate learning schemes to solve the problem of DOA estimation of spatially adjacent coherent sources within a beamwidth. Among them, the introduction of angle separation reduces the computation of traditional two-dimensional search, and the model can maintain high performance in some harsh environments. Therefore, it is also our motivation to build a more suitable learning model by mining deeper data features through coherent signals.

However, based on the existing neural network methods, there are still the following problems in coherent DOA estimation: (1) When the signal environment changes, that is, the training parameters such as signal-to-noise ratio and number of snapshots, are not consistent with the test parameters, the estimation accuracy will decline under the new signal environment. So, the neural network needs a large number of samples to learn again. (2) To improve the estimation accuracy, the quantization angle step needs to be relatively small, which leads to an increase in the amount of search, thus greatly increasing the computational complexity. (3) When the quantization angle step based on the (2) problem is relatively small, there will be some errors for the neural network to solve the long sequence, which will affect the final DOA results. In order to solve the above problems that will be faced, we can give the corresponding preliminary scheme, that is, we can solve the (1) existing problem by using meta−reinforcement learning through a small sample set to obtain the angle interval of the incident signal, and then complete the two-dimensional to one-dimensional spectral peak search through its angle interval, and then solve the (2) existing problem, and finally get the DOA results. Therefore, this paper proposes a small sample meta reinforcement learning method based on Markov Decision Process (MDP) to improve DOA based on coherent angle interval feature. It is intended to model the decision process of the angle interval feature sequence of coherent DOA of meta−reinforcement learning method as MDP, and then the target output can be weighted by the attention mechanism of the sequence to sequence (S2S) network. This will affect the selection of context information of small sample data sets to improve the accuracy of the output long sequence decoder; that is, the probability output of solving each angle interval in the angle interval feature vector sequence will be improved to solve the (3) existing problem, and then complete the whole machine translation process.

## 2. MDP Model for Coherent DOA Estimation

### 2.1. Quadratic Feature Extraction of Coherent DOA Estimation

Let the incoming wave direction of the coherent signal $s_{d}\left(t\right),{\;s}_{i}\left(t\right)$ be $\theta_{d},{\;\theta }_{i}$, respectively, and its time domain waveform, $s_{d}\left(t\right),{\;s}_{i}\left(t\right)$, is the sampling moment where there are T sets of snapshots. In order to estimate the coherent DOA, the angle quantization is carried out in the interval range of the possible incoming wave direction, and the quantization unit is Δϕ. There is also the discrete direction set after quantization, where $\emptyset =\left[\emptyset_{1},\emptyset_{2},\ldots ,\emptyset_{l},\ldots ,\emptyset_{L}\right]$, and where where $\Delta \phi =\emptyset_{l+1}-\emptyset_{l}$. In each quantization direction, the signal is represented as $\bar{s_{l}}\left(t\right)$, where l=1…L. Then, the output of the antenna array of M unit $x\left(t\right)\in {\mathbb{C}}^{M\times N}$ can be expressed as follows:

(1) $$x\left(t\right)=\sum_{l=1}^{L}d(\emptyset_{l})\bar{s_{l}}\left(t\right)+n\left(t\right)\;t=1,2\ldots ,T$$

In the above equation, $d\in {\mathbb{C}}^{M\times 1}$ is the guide vector, L is the quantization number, and $n\left(t\right)={\left[n_{1}\left(t\right),n_{2}\left(t\right),\ldots ,n_{M}\left(t\right)\right]}^{Τ}\in {\mathbb{C}}^{M\times N}$ denotes White Gaussian Noise with power $\sigma_{n}^{2}$. Obviously, when the time of Δϕ is enough, the coherent incoming waves $\theta_{d}$ and $\theta_{i}$ can be approximated to the quantization line angle.

(2) $$\bar{s_{l}}\left(t\right)=\{\begin{array}{c} s_{d}\left(t\right),\;\;\;\left|\theta_{d}-\theta_{l}\right|<\frac{1}{2}\Delta \emptyset \\ s_{i}\left(t\right),\;\;\;\left|\theta_{i}-\theta_{l}\right|<\frac{1}{2}\Delta \emptyset \\ 0 \end{array}$$

In the following, the quadratic spatial spectral characteristics [16] of **R** can be derived from the observations of the covariance matrix **R** of the array output x(t). Let **R** be the covariance matrix of x(t),

(3) $$R=E[x\left(t\right)x^{H}\left(t\right)]=\sum_{l=1}^{L}(\eta_{l}d\left(\emptyset_{k}\right)d^{H}\left(\emptyset_{k}\right))+\sigma_{n}^{2}I$$

Define $\eta_{l}$ to denote the signal power in the quantization direction,

(4) $$\eta_{l}=E\left[\bar{s_{l}}\left(t\right){\;s\;¯}_{l}^{H}\left(t\right)\right]$$

Here, (.)H denotes the Hermitian matrix, E[.] is the expectation and I is the unit matrix.

According to the above equation and definition, the quadratic characteristic space spectrum η of R can be obtained as follows:

(5) $$\eta \approx {\tilde{D}}^{H}y=[\eta_{1},\eta_{2}\ldots ,\eta_{L}]$$

Among thrdr, $\tilde{D}=\left[D_{1};D_{2};\ldots ;D_{M}\right]$, $D_{m}\left(:,k\right)=d\left(\emptyset_{k}\right)d^{H}\left(\emptyset_{k}\right)e_{m}$, and $e_{m}$ is a M×1 dimensional column vector with the mth element being 1 and the remaining elements being 0. M is the first number of the array, while $y=\left[y_{1};y_{2};\ldots ;y_{M}\right]$, $y_{m}$ is the mth column of the covariance matrix R. In this paper, the spatial spectral characteristic sequence will be transformed into an embedding vector and input to the neural network subsequently.

In order to avoid a two-dimensional search of the network output when solving the coherent DOA, the quadratic characteristics of the coherent angular interval feature vector are further extracted based on the above discrete set of quantized directions. The variation interval of the coherent angular interval quantities is [Δ∅, (L−1)Δ∅]. Therefore, the set of coherent angular interval feature vectors λ can be expressed as follows:

(6) $$\lambda =\left[\lambda_{1},\lambda_{2},\ldots ,\lambda_{k},\ldots \lambda_{L-1}\right]\;\mathrm{And}\;\lambda_{k}=k\Delta \phi \;,\;k=1,2\ldots L-1.$$

The problem is equivalent to inputting a sequence feature η of the length L, finding the sequence feature λ of the length L−1, and finally converting the solution based on λ to a one-dimensional peak search to find the DOA.

### 2.2. MDP Model for Coherent DOA Estimation

When the quantization unit Δ∅ is smaller, the error amount of signal $\bar{s_{l}}\left(t\right)$ in each quantization direction is smaller, and the length of the corresponding angular interval feature vector sequence λ is also longer. Therefore, machine translation technology is needed to indirectly obtain the output of the angular interval feature vector using the information of the angular interval feature vector output obtained in the λ in front of the paper, so as to complete a series of prediction work, and therefore more samples are needed to complete a more accurate prediction process in the face of such long sequences. Here in this paper, the probability output of each angle interval of λ in the sequence corresponds to each task in the text, and by using the contextual information of each task in the sequence with a small number of samples, the accuracy of each step of prediction can be further improved. Therefore, this paper models the sequence decision process of coherent DOA estimation as MDP [24] (Markov decision process), while setting the quadratic characteristic space spectrum η as the input of the S2S network, followed by the probabilistic output of each angular interval in λ in which the sample will be in different states $s_{1},{\;s}_{2},\ldots$ at different moments, so the different state transition processes in a time corresponds to an MDP process. This MDP process can be defined by a quintet {S,A,P,R,γ}, where S is the state space, A is the action space, P is the state transfer matrix, R is the state transfer gain, and γ is the forward gain discount. Allow that DOA intelligence will act η as a signal environment, and in this environment find exactly the angular interval between signals in a sequence λ of angular interval feature vectors of length 1 to L−1 with maximum probability, i.e., η input by S2S network is complex and variable, while η makes decisions about giving the corresponding action $a_{k}$ according to the environment, where the action $a_{k}$ is represented by a binary 0 or 1, respectively, representing the presence or absence of a certain angular interval in the sequence λ. In order to obtain the optimal strategy, the DOA intelligence needs to obtain more gain for each action executed, i.e., when the sequence of actions is executed so that the total gain is maximized. The action $a_{k}$ and the network input η form a new state $s_{k}$ to predict the next action $a_{k+1}$, and finally the probability output of each angle interval in the sequence λ is obtained in this form.

#### 2.2.1. Definition of the State Space S

When the MDP model is in a certain state, the DOA intelligence makes a decision to execute the corresponding action according to the changes in the signal environment and moves to a new state after executing the action. The MDP model learns through the neural network to form the optimal decision to execute the best action.

The input sequence of the neural network is η. Each time the action $a_{k}$ is predicted by the neural network, the combination of action $a_{k}$ and η form a new state [25], which is used as the input state for the network to predict the action $a_{k+1}$.

Allow that the DOA intelligence has executed the first k actions to obtain the action sequence $a_{k}$, k=1,2…k. As $A_{1:k}$ = [$a_{1},a_{2},\ldots ,a_{k}]$, the set of states $S≔\{s_{k}|s_{k}=\left(\eta ,A_{1:k}\right)$}, k = 1, 2 ⋯, L−1 in the prediction process. State $s_{k}$ consists of the spatial spectrum η of the array auto-correlation matrix and the set of the first k actions, and the full set of these states forms the state space S:

(7) $$S≔\{\left(\eta ,A_{1:k}\right)|A_{1:k}=\left[a_{1},a_{2}\ldots ,a_{k}\right]\},\;k=1,\;2\ldots ,\;L-1$$

#### 2.2.2. Definition of Action Space A

The output sequence of the neural network is a sequence of coherent angular interval feature vectors, i.e., $\lambda =\left[\lambda_{1},\lambda_{2},\ldots ,\lambda_{k},\ldots \lambda_{L-1}\right]$. To identify the presence of coherent angular interval features of the incoming wave, dual classification decisions of $a_{i}$ = 0 or 1 are required for the execution action of the output sequence, which is a binary classification decision problem.

When the first k task decision is 1, i.e., $a_{k}$ = 1, it indicates the presence of coherent angular interval feature $\lambda_{k}$ in the incoming wave with the value kΔϕ. When there are more than one task decision of 1, it means that there are more than one coherent angular interval features. When the kth task decision is 0, i.e., $a_{k}$ = 0, it means that there is no coherent angular interval feature $\lambda_{k}$. Thus, the coherent DOA estimation problem is transformed into a decision problem with a sequence of L−1 tasks, $a=\left[a_{1},a_{2},\ldots ,a_{L-1}\right]$, and the sequence decisions constitute the action space A:

(8) $$A≔\{a_{k}|a_{k}\in \left\{0,1\right\}\},\;k=1,2\ldots ,L-1$$

#### 2.2.3. Definition of State Transfer Gain

State transfer of a system describes the effect between adjacent actions of sequential decisions. The state in which a DOA intelligence is in changes after it executes an action. Therefore, it is required that the neural network gradually learns the optimal strategy so that it can perform the best action. The strategy corresponds to the parameters of the neural network. Let the parameters of the neural network be w. In order to obtain the optimal strategy, it is necessary to obtain more gains for each action execution and the total gain obtained maximum after the execution of the sequence of actions. To avoid local optimality, the total gain of state transfer is defined as the sum of identification errors in coherent angle interval feature of the sequence.

The goal of sequential decisions making is to minimize the DOA estimation error, and in this paper, the total gain of each MDP state transfer process is defined as the negative value of the cross-entropy loss function:

(9) $$\mathrm{Loss}=\frac{1}{N}\overset{N}{\underset{i=1}{\sum }}l_{i}=-\frac{1}{N}\overset{N}{\underset{i=1}{\sum }}(b_{i}\log\left(p_{i}\right)+\left(1-b_{i}\right)\log\left(1-p_{i}\right)$$

Here $b_{i}$ denotes the label of output $a_{i}$ with positive class 1 and negative class 0. $p_{i}$ is the probability that output $a_{i}$ is predicted to be 1, and N is the total number of sequence tasks = (L−1)* number of samples.

## 3. DOA Estimation Based on S2S Network Meta−Reinforcement Learning Algorithm

After solving the MDP model, the probability output of each angle interval in the coherent angle interval feature vector sequence λ can be obtained successively. At the same time, in order to improve the estimation accuracy, the quantitative angle step should take a smaller value, and thus a long sequence λ will be obtained. In order to store the encoding information of the long sequence and make full use of the information association between the output sequence terms to explore the unknown number of signals efficiently, this paper uses S2S (sequence to sequence) deep neural network for the probability output of each angle interval in λ corresponding to where the decisions consisting of each task in the text are solved.

According to the definition of the sequential state transfer process, after each task in the sequence is predicted, a subset of the predicted tasks will form a new state together with the network input η and then predict the next task in the sequence. S2S neural network needs to be learned by the DOA intelligence in order to obtain the best decision, and the network parameters are iterated and updated until the global loss function converges, i.e., when the total gain of each MDP state in transfer process is maximum, the global loss reaches a minimum.

Since the coherent signal can form an arbitrary coherent signal environment under different influences such as coherence coefficient, signal-to-noise ratio, and number of snapshots, the environment is relatively complex. Therefore, in order to avoid the neural network relearning deeply every time a new signal environment appears, this paper will use a meta−reinforcement learning algorithm to iterate the network strategy and carry out the optimization of the S2S network parameters.

### 3.1. Expression of S2S Deep Neural Network

**Figure 1:**
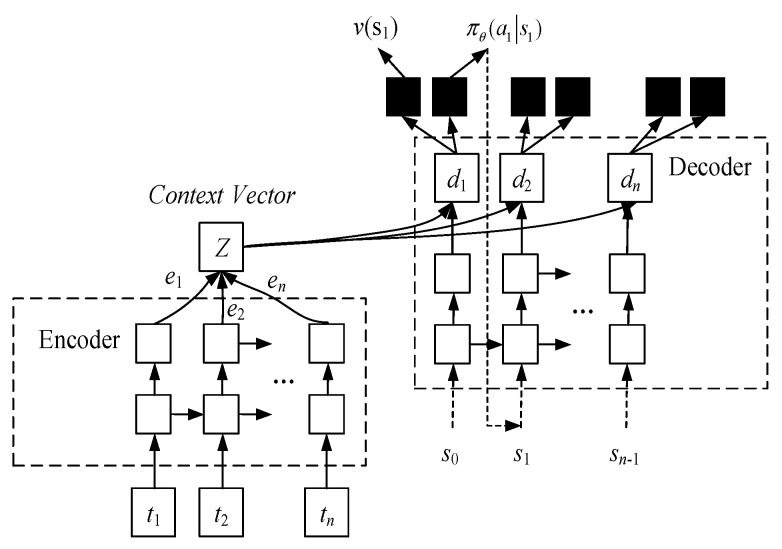
S2S Neural Network Framework.

The S2S neural network [26] consists of an encoder and a decoder, both of which are RNN network (Recurrent Neural Network), as shown in Figure 1. Let the parameters of the neural network be w, then when the input state is s, the conditional probability of outputting the best decision can be rewritten $\pi_{w}(a|s)$. According to the input $t_{i}$, the neural network is first encoded by the encoder for learning and memory, after which the output $d_{j}$ is passed through the decoder according to the memory of the network, and then passed through two different activation functions, corresponding to the output state value function v(s) and the decision sequence ${\mathrm{probability}\;\pi }_{w}(a|s)$, respectively.

Denoting the encoder and decoder as $f_{\mathrm{enc}}$ and $f_{\mathrm{dec}},$ respectively, the output of the encoding part is as follows:

(10) $$e_{i}=f_{\mathrm{enc}}\left(t_{i},e_{i-1}\right)$$

The output of the decoding part is as follows:

(11) $$d_{j}=f_{\mathrm{dec}}\left(z_{j},s_{j-1},a_{j-1}\right)$$

(12) $$Z_{j}=\overset{n}{\underset{i=0}{\sum }}\alpha \mathrm{jiei}$$

(13) $$\alpha \mathrm{ji}=\frac{\exp\left(\mathrm{score}\left(\mathrm{dj}-1,\mathrm{ei}\right)\right)}{\sum_{k=1}^{n}\exp\left(\mathrm{score}\left(\mathrm{dj}-1,\mathrm{ek}\right)\right)}$$

The output sequence of the decoder does not correspond one-to-one with the input sequence of the encoder, but predicts the next task based on the prediction result and the context of the previous task. The input of the decoder consists of three parts, including the output weighted sum $z_{j}$ of the encoder, and the result of the decision execution of the previous step $s_{j-1}{\;\mathrm{and}\;a}_{j-1}$. $z_{j}$ is the context of the jth decoder step, implying the properties of the input data.

At the same time, when the sequence is very long, in order to avoid the difficulties for S2S neural network to learn reasonable output representation, the attention mechanism is introduced into the S2S neural network [27] to make weighted changes to the target output, thus affecting the selection of context information.

The input sequence increases the start marker to start as the initial value of the decoding, and the number of iterations stops when the last stop marker of the input sequence is encountered. In the S2S neural network architecture, the output vector of the decoder is a dimensional vector d of n = L−1, which is passed through the softmax output layer and fully connected output layer, respectively, to obtain the n-dimensional strategy function $\pi_{w}$ and value function v($s_{i})$. The strategy function $\pi_{w}$ corresponds to the probability that the decision action takes a certain a, and its sum is 1. The decision action $a_{j}={\mathrm{argmax}}_{a}\left(\pi_{w}\right)$ of the jth step is obtained by the greedy algorithm.

### 3.2. The Optimal Strategy Algorithm Based on Meta−Reinforcement Learning

In order to obtain the optimal decision sequence so that the error sum of coherent angular interval feature vector sequence λ obtained is minimized, the S2S neural network needs to be learned and the network parameters updated to global convergence. To be able to use the prior model to avoid relearning deeply when the coherent DOA estimation encounters a new signal environment requires a large number of samples to train the network. Therefore, the meta−reinforcement learning algorithm [28] is used to train the parameters of the S2S neural network in this paper.

Meta−learning, also known as learning to learn, is a method for solving few-shot learning by using previous knowledge and experience to guide the learning of new tasks and equipping the network with the ability to learn. Existing in deep learning is to first tune the parameters artificially, and then directly train a deep model for a specific task. Meta−learning, on the other hand, first trains a better hyper-parameter through other tasks and then trains for a specific task. Meta−learning hopes to make the model acquire an ability to learn tuning parameters so that it can quickly learn new tasks based on the acquisition of existing knowledge.

This proposed solves the coherent DOA estimation based on the MAML meta−learning algorithm [29], and after training multiple MDP processes in an outer loop to obtain hyper-parameters, the hyper-parameters are used as initial values for a specific MDP process to train the S2S network. By iterating over each other, such that a fast convergence of the S2S network strategy can be achieved with a small number of samples when a new environment arises, as shown in Algorithm 1 is solved iteratively in order to obtain the optimal decision sequence and thus the optimal action sequence.
**Algorithm 1** The Optimal Strategy Training Algorithm Based on Meta−reinforcement Learning1: Given a coherent DOA angular interval feature vector sequence task distribution ρ(T). 2: Randomly initialize the meta−reinforcement learning parameter w. 3: Outer loop: for i∈{1,…,n} do. $4:\;\mathrm{Collect}\;n\;\mathrm{sequences}\;\mathrm{of}\;\mathrm{coherent}\;\mathrm{angular}\;\mathrm{interval}\;\mathrm{feature}\;\mathrm{vectors}\;\left\{T_{0},T_{1},\ldots ,T_{n}\right\}$ from ρ(T). $5:\;\mathrm{Inner}\;\mathrm{loop}\;\mathrm{for}\;\mathrm{each}\;\mathrm{coherent}\;\mathrm{angular}\;\mathrm{interval}\;\mathrm{feature}\;\mathrm{vector}\;\mathrm{sequence}\;T_{i}$, do. 6: $\mathrm{Initialize}\;\mathrm{the}\;S2S\;\mathrm{network}\;\mathrm{parameters}\;w_{k}^{0}$=w$\;\mathrm{corresponding}\;\mathrm{to}\;\mathrm{this}\;\mathrm{sequence}\;\mathrm{task},\;i.e.,\;\mathrm{by}\;\mathrm{assigning}\;\mathrm{the}\;\mathrm{parameters}\;\mathrm{obtained}\;\mathrm{from}\;\mathrm{the}\;\mathrm{outer}\;\mathrm{loop}\;\mathrm{to}\;\mathrm{the}\;\mathrm{inner}\;\mathrm{loop}\;\mathrm{and}\;\mathrm{saving}\;\mathrm{the}\;\mathrm{optimal}\;\mathrm{value}\;w_{k}=w$ of the network parameters. $7:\;\mathrm{The}\;\mathrm{trajectory}\;\mathrm{sequence}\;D=\left(\tau_{1},\tau_{2},\ldots ,\;\right)\;\mathrm{of}\;\mathrm{the}\;\mathrm{kth}\;\mathrm{task}\;\mathrm{of}\;\mathrm{the}\;\mathrm{sequence}\;\mathrm{is}\;\mathrm{collected}\;\mathrm{using}\;\mathrm{the}\;\mathrm{sampling}\;\mathrm{strategy}\;\pi_{w_{k}^{0}},\;i.e.,\;\mathrm{the}\;\mathrm{Adam}\;\mathrm{gradient}\;\mathrm{update}\;\mathrm{trajectory}\;\mathrm{of}\;\mathrm{the}\;\mathrm{kth}\;\mathrm{angular}\;\mathrm{interval}\;\mathrm{feature}\;\mathrm{of}\;\mathrm{the}\;\mathrm{sequence}.\;\mathrm{The}\;S2S\;\mathrm{network}\;\mathrm{parameters}\;\mathrm{are}\;\mathrm{computed}\;\mathrm{on}\;D.\;\mathrm{Based}\;\mathrm{on}\;\mathrm{the}\;\mathrm{initial}\;\mathrm{value}\;\mathrm{of}\;\mathrm{the}\;\mathrm{outer}\;\mathrm{loop}\;\mathrm{strategy},\;\mathrm{the}\;\mathrm{task}\;\mathrm{quickly}\;\mathrm{obtains}\;\mathrm{the}\;\mathrm{convergence}\;\mathrm{value}\;w_{k}^{\prime }=w_{k}+\alpha \nabla_{w_{k}}J_{T_{k}}\left(w_{k}\right)\;\mathrm{of}\;\mathrm{its}\;\mathrm{policy}\;\mathrm{after}\;\mathrm{only}\;u\;\mathrm{iterations},\;\alpha$ being the inner MDP learning rate. 8: After each task of the sequence converges, the system state is updated and the strategy learning for the next task proceeds. The parameters of the convergence strategy corresponding to each task of this sequence are saved. 9: End for. 10: The saved network parameters learned from the previous sequence are passed to the outer loop to train the hyper-parameters. 11: $\mathrm{The}\;\mathrm{outer}\;\mathrm{loop}\;\mathrm{performs}\;\mathrm{learning}\;\mathrm{of}\;\mathrm{the}\;\mathrm{priori}\;\mathrm{hyper}-\mathrm{parameters}\;\mathrm{and}\;\mathrm{updates}\;\mathrm{the}\;\mathrm{parameter}\;w=w+{\beta g}_{t}\left(w_{i}^{\prime }\right)$$\;\mathrm{using}\;\mathrm{the}\;\mathrm{gradient}\;g_{t}$. β is the outer learning rate, which is a balanced parameter for exploration and exploitation. 12: End for.

J and $\nabla_{w_{i}},$ in the above algorithm, are the loss function and gradient operator of the S2S network, respectively. The action of the MDP model is essentially a binary classification problem, labeled [0, 1], while the S2S network outputs a probability value through the sigmoid output layer, which reflects the probability that the prediction is 1, $\hat{a}\;=p(a=1|s)$. Therefore, in order to characterize the gap between the predicted output and the true value, the cross-entropy function (Equation (9)) is used to define the loss function. The more consistent the predicted output ${\hat{a}}_{k}$ is with $a_{k}$, the smaller the loss value is, which means the greater the gain of the state transfer process of the MDP.

The term $g_{t}$ is the second-order gradient operator for meta−reinforcement learning, and when maximizing the second-order operator $g_{t}$ by the Adam gradient [30] ascent method, it is the gradient of the gradient that leads to too much computational complexity, so the first-order value is used to approximate $g_{t}$, see Equation (14),

(14) $$g_{t}=\frac{1}{N}\overset{N\;}{\underset{i=1}{\sum }}[(w_{i}^{\prime }-w)/\alpha /u]$$

The term w  is the training parameters of the outer loop network, output to the inner loop. The term α is the learning rate of the inner loop, and u is the number of Adam gradient descent of the inner loop; N is the number of sequences of the outer loop, and $w_{i}^{\prime }$ is the set of convergence values of the network parameters of the ith sequence of the inner loop, output to the outer loop to participate in the calculation of the second-order gradient operator.

When the signal environment changes, based on the strategy learned in the outer loop, the inner loop converges quickly and completes training in just a few steps, i.e., a strategy is found that generates a good adaptation to the new task $T_{i}$ by a few gradient steps, a meta−strategy $\pi_{w}$ with strong generalization capability is maintained, and only a few gradient descent steps are needed to significantly improve the performance of the model on a previously unseen task $T_{i}$. After the test signal is input to the neural network with optimal parameters, the angular interval λ in the coherent angular interval feature vector sequence λ can be found, assuming that λ is at the maximum position in λ, we can obtain $\theta_{i}=$ $\theta_{d}-\lambda$, which translates into a one-dimensional peak search to find the DOA. Here, define $\alpha_{d}=[\alpha_{1},\alpha_{2},\ldots ,\alpha_{i},\ldots ,\alpha_{L}]$ the over-perfect set of $\theta_{d}$ and $\beta_{i}=[\beta_{1},\beta_{2},\ldots ,\beta_{i},\ldots ,\beta_{L}]$ the over-perfect set of $\theta_{i}$, and $\beta_{i}$ = $\alpha_{i}-\lambda$. The mth column of R can be written as follows:

(15) $$y_{m}^{\prime }=D_{m}^{\prime }\eta +\sigma_{n}^{2}e_{m}$$

and

(16) $$D_{m}^{\prime }\left(:,k\right)=\left(d\left(\alpha_{k}\right)d^{H}\left(\alpha_{k}\right)+d\left(\beta_{k}\right)d^{H}\left(\beta_{k}\right)\right)e_{m}$$

$\theta_{d}$ can be obtained by the following equation:

(17) $$\hat{\theta_{d}}=\arg\underset{\alpha_{d}}{\max}{\tilde{D}}^{\prime H}y^{\prime }$$

where ${\tilde{D}}^{\prime }=\left[D_{1}^{\prime };D_{2}^{\prime };\ldots ;D_{M}^{\prime }\right]$, $y^{\prime }=\left[y_{1}^{\prime };y_{2}^{\prime };\ldots ;y_{M}^{\prime }\right]$. Finally, by searching the maximum value of the spatial input spectrum η in space, the corresponding $\theta_{d}$ and $\theta_{i}$ are obtained, respectively.

## 4. Experiments and Analysis of Results

The parameters used in the experiments include MDP model parameters, S2S network parameters, DOA signal, and array parameters. The simulation parameters are shown in Table 1.

Consider an M = 21, λ = 1 m, and a ULA with an array element spacing of 0.5 m to verify the performance of S2S-MRL. The performance of this method in terms of SNR and DOA accuracy of the number of snapshots is verified by experiments on two coherent signals. Considering that the beam width is about 5 °, within the range of [0°, 3°] and [−3°, 0°], the angle of the first signal direction $\theta_{1}$ and the angle of the second signal direction $\theta_{2}$ are randomly generated. The angle interval of the training data set is randomly distributed between [0°, 6°], 150000 data are used for training, and another 3000 data are used for verification. The classic physically driven SSMUSIC and OGSBL algorithms and the data-driven algorithms in literature [15,16,23] are compared with the methods in this paper. The neural network training process is based on MATLAB 2021b and the Adam optimizer, while all experiments are performed on a computer with an 12th Gen Intel (R) Core (TM) i7-12700H 2.30 GHz and an NVIDIA GeForce RTX 3090. In this process, the dropout strategy is used to prevent over-fitting. The dropout ratio is 0.95.

**Figure 2:**
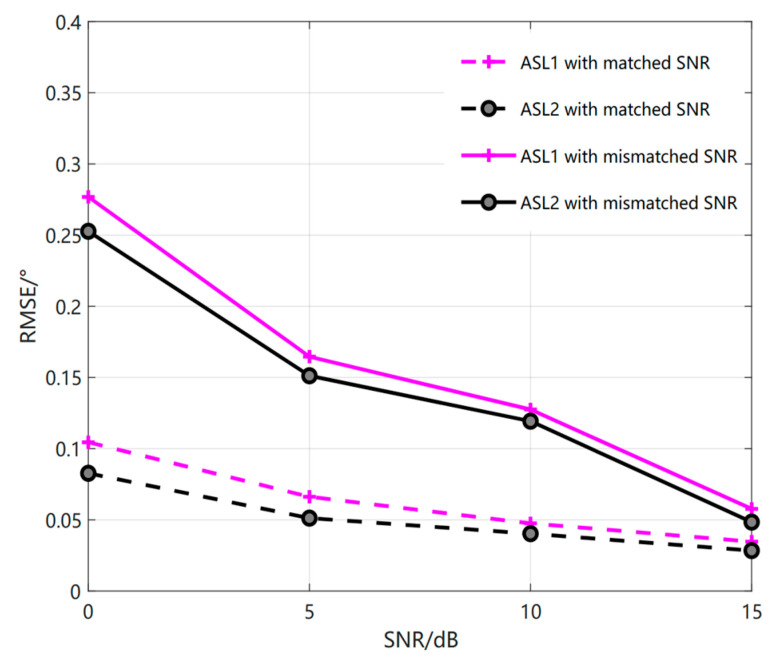
Relationship curve between RMSE and SNR (The real line is mismatched, and the imaginary line is matched.).

**Figure 3:**
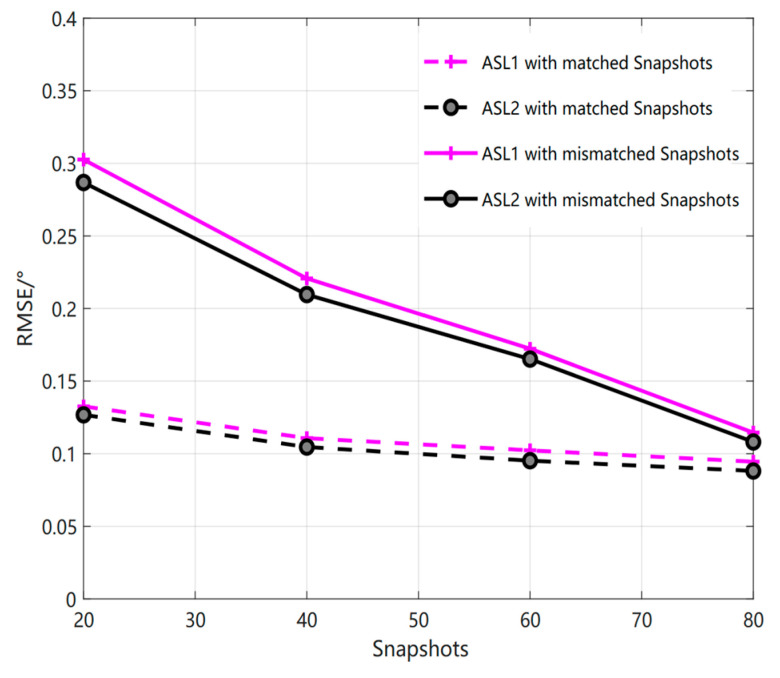
Relationship curve between RMSE and Snapshots (The real line is mismatched, and the imaginary line is matched.).

We have carried out the simulation of ASLs method in literature [23] for mismatched scenes with large differences in SNR and snapshot number, as shown in Figure 2. That is, when the training SNR is 20 dB, the test SNR is 0 dB, 5 dB, 10 dB, and 15 dB, respectively, in which the minimum difference of the signal-to-noise ratio in the case of mismatch is 5 dB and the maximum difference is 20 dB. We can see that the estimation accuracy will be affected when the difference between the training and test SNR is more than 10 dB. For example, when the signal-to-noise ratio of ASL1 method is 20 dB, that is, when the signal-to-noise ratio is 0 dB, the RMSE value is 0.1724°, and under the same conditions, the RMSE value of ASL2 method is 0.17°. In the same way, when the number of snapshots does not match, that is, when the number of training snapshots are 100, the number of test snapshots are 20, 40, 60, and 80, respectively. The minimum difference between the number of snapshots in the case of mismatch is 20 and the maximum difference is 80. From Figure 3, we can see that the estimation accuracy will also be affected when the number of snapshots differs by more than 40. When the environmental difference gradually increases, the performance of the estimation accuracy will also become relatively poor compared with that of the previous matching. For example, when the difference in the number of snapshots of ASL1 method is 80, that is, when the number of snapshots is 20, the difference in RMSE value is 0.1679°, and under the same conditions, the difference in RMSE value of ASL2 method is 0.1692°. Therefore, from the above figure, we can see that the accuracy of the ASLs method for coherent DOA in this new environment with large differences has been greatly affected. Therefore, under the condition of such large difference environment, the ASLs method may need to be retrained to have a better estimation result in the test stage.

### 4.1. Loss Function Trained under Different Influences

The simulation parameters are set as incoming wave angle range [−3°, 3°], SNR = 5 dB, and Snapshots = 40 to conduct the experiments under different quantization influences. Firstly, the meta−knowledge experience is obtained from the meta−training phase under the first training parameter, and secondly, based on the meta−knowledge experience obtained from the meta−training phase, effective generalization training is performed in the meta−testing phase using a small number of samples to complete the experiments under different influences as follows.

**Figure 4:**
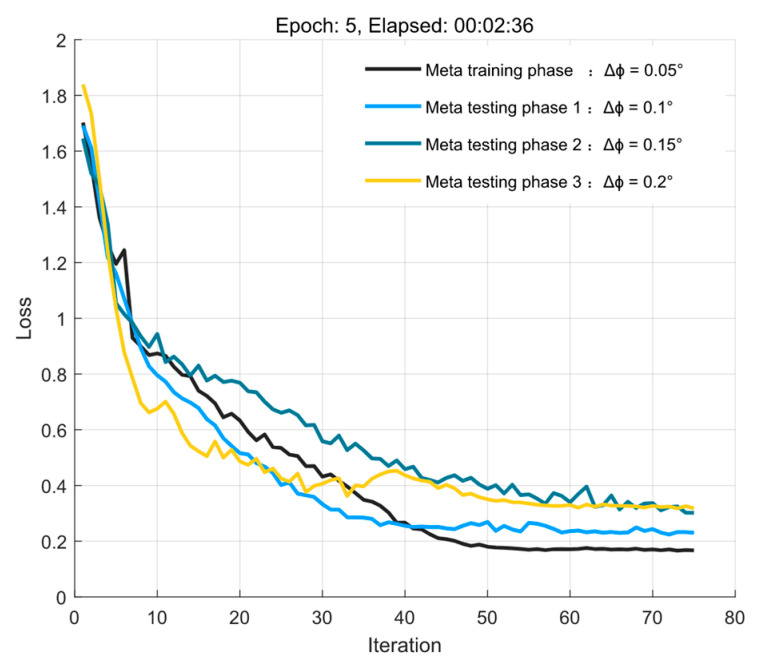
Loss convergence curve when taking different quantization.

Since the quantization unit Δϕ directly affects the length of the sequences, the experiments are conducted with different training parameters Δϕ = [0.05°, 0.1°, 0.15°, 0.2°] for the quantization units, respectively, and the input sequence length L = 121 and the output sequence length L−1 = 120 when Δϕ is 0.05. The experimental results are shown in Figure 4. The S2S network model based on the knowledge experience obtained under the first training parameter in the meta−training phase still maintains stable performance for different sequence lengths in the meta−testing phase, and basically starts to converge after 20 iterations. Since the MDP model takes into account the influence between similar tasks in the sequence, the impact of the error from quantization in the meta−test phase is mitigated when Δϕ takes different values.

**Figure 5:**
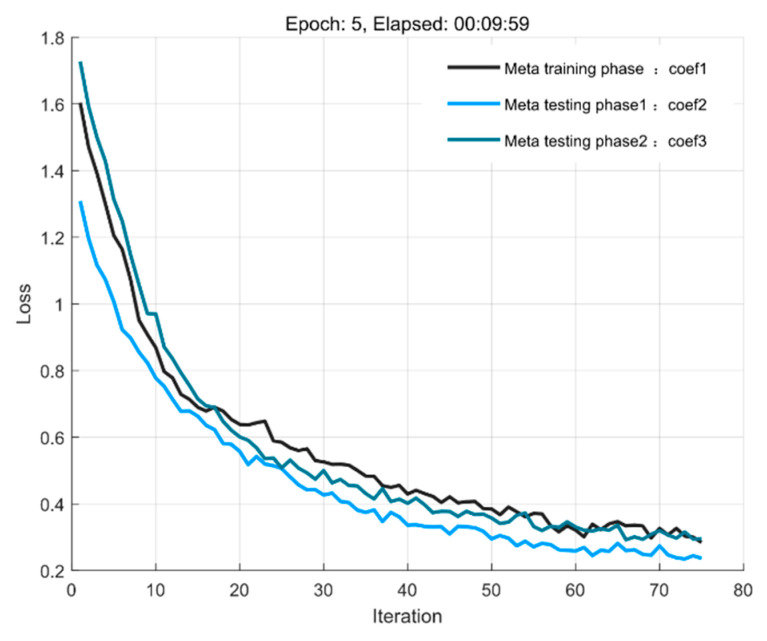
Loss convergence curves for sources with different.

Since the correlation strength between coherent sources has an impact on DOA identification, the paper extracts the quadratic spatial spectral features of the auto-correlation array as the input of the S2S network with simulation parameters set to the incoming wave angle range of [−3°, 3°], SNR = 5 dB, Snapshots = 40, Δϕ = 0.1° to conduct experiments under the influence of different coherent coefficients. Figure 5 takes any two items in the set of correlation coefficients at a time to form coherent sources, and the results of this experiment as shown in Figure 5 can show that the feature sequence is suitable for DOA detection of coherent sources. It can also be seen that the model is not influenced by the correlation strength between coherent sources in the meta−testing phase, thus changing the trend of training loss convergence.

**Figure 6:**
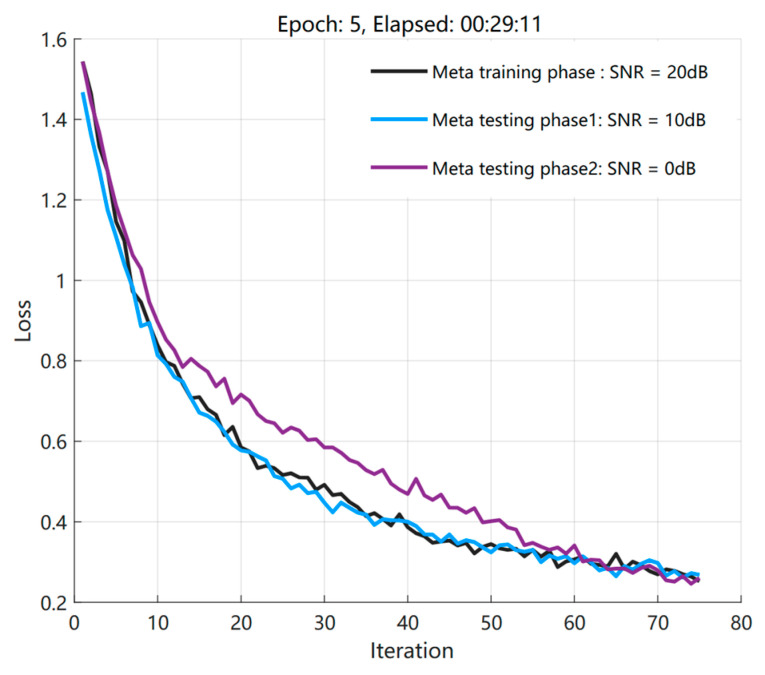
Loss convergence curves under different SNR environments.

Here, considering the generalization ability of S2S neural-network meta−reinforcement learning algorithm from high SNR to low SNR, the simulation parameter is set as the range of incoming wave angle is [−3°, 3°], snapshots = 40, Δϕ = 0.1°, and the experiment is carried out under the influence of different signal to noise ratio environment. Finally, the experimental results are shown in Figure 6. After obtaining meta−knowledge experience from 20 dB samples in the meta−training stage, its experience is used in the meta−testing stage to generalize the training of models with a difference of 10 dB and 20 dB with a small number of samples, that is, the training of 10 dB and 0 dB models. At the same time, the figure can clearly show the meta−reinforcement learning algorithm, and can effectively adapt to the new signal environment, that is, the new sequence based on the prior knowledge obtained under the training of multiple sequence samples can also quickly converge.

**Figure 7:**
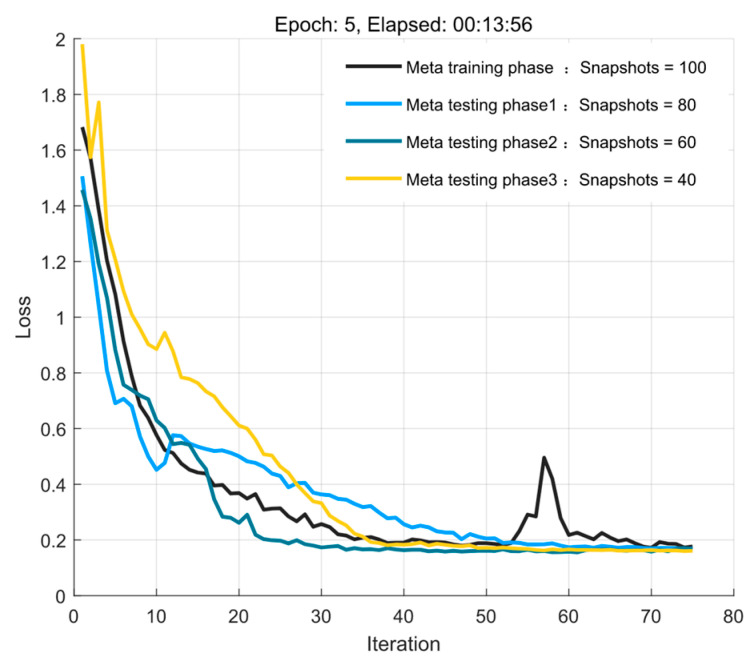
Loss convergence curves under different snapshot environments.

At the same time, considering the generalization ability of the S2S network meta−reinforcement learning algorithm from high to low snapshots, the simulation parameters are set to [−3°, 3°], SNR = 5 dB, Δϕ = 0.1° for the experiments under the influence of different snapshot environments. The experimental results are shown in Figure 7. The experiments are the same as the above experiments, in which a certain amount of experience is gained from the samples with Snapshots = 100 in the meta−training phase, and then a small number of samples are used in the meta−testing phase of the same distribution to quickly generalize the model for the three new environments with snapshots = 80, 60, and 40, respectively. At the same time, Figure 7 shows that in the meta−training phase, there is an obvious unstable upward trend of the loss value between 50 and 60 iterations, but the algorithm allows this search for the optimal strategy to jump out of the local optimal solution and then find the global optimal solution quickly, i.e., the global optimal strategy.

**Figure 8:**
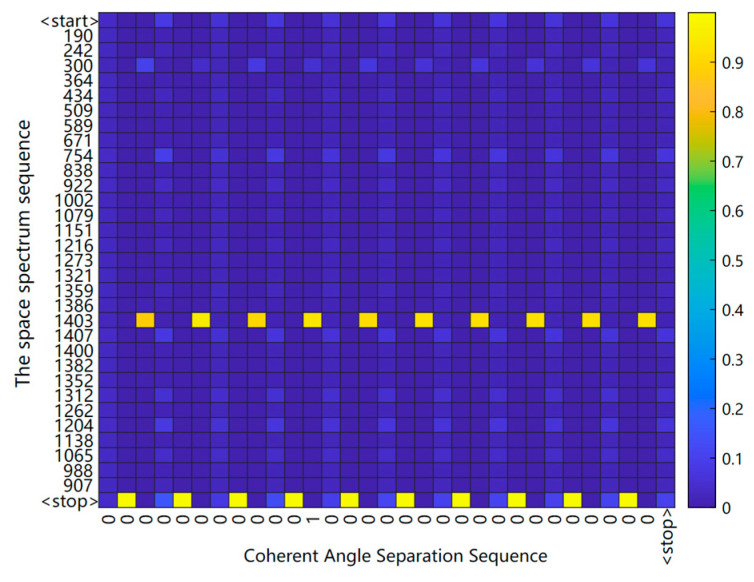
Heat map of attention.

When the input sequence of the S2S network is long, it is difficult to retain all the necessary information, so the probability of generating each task item in the output sequence depends on which important task items are selected in the input sequence. Figure 8 shows the task items that are attended to in the sequence during decoding, and the vertical coordinates are the encoded values of the input.

### 4.2. Performance Testing in the Original Environment Based on the Meta−Training Phase

**Figure 9:**
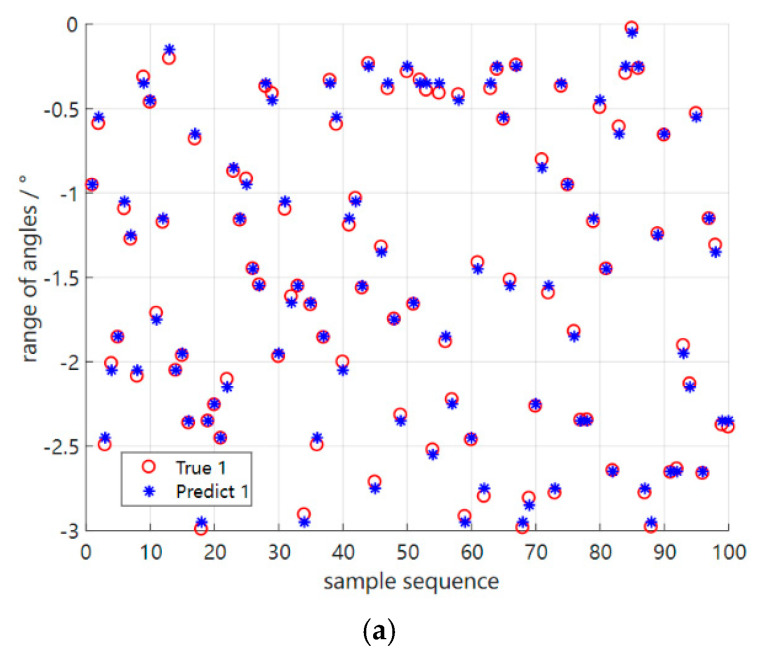

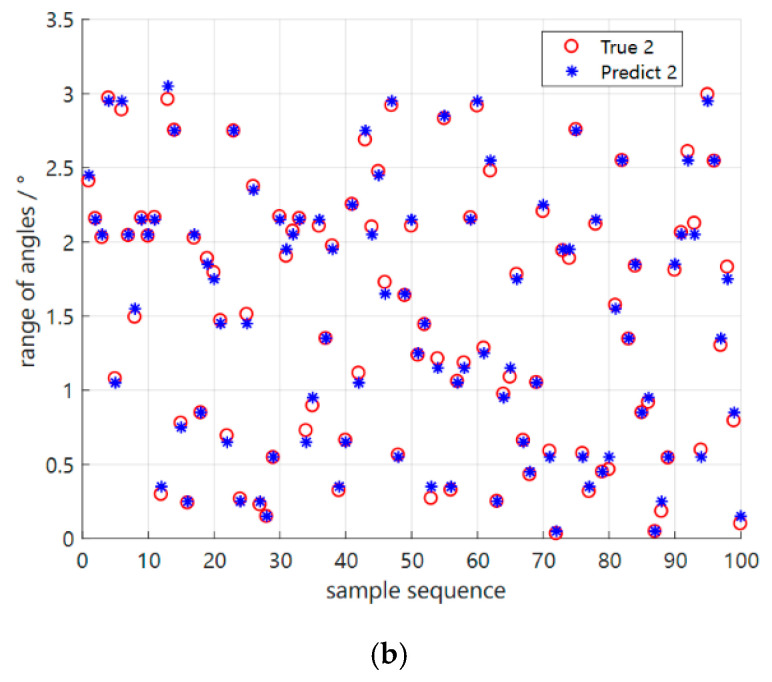
Error plots of the S2S network meta−reinforcement learning algorithm based on two perspectives in the original environment, respectively (**a**,**b**). (**a**) Error plot of the S2S network meta−reinforcement learning algorithm based on the first angle in the original environment. (**b**) Error plot of the S2S network meta−reinforcement learning algorithm based on the second angle in the original environment.

**Figure 10:**
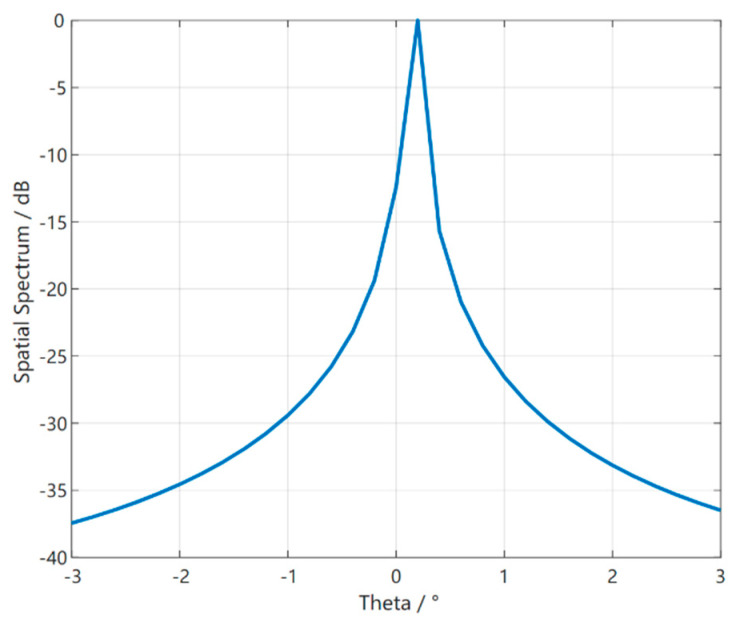
Detection results of Coherent DOA Classical Dimension Reduction Method.

In the experiments, given different two coherent sources, Figure 9 uses the S2S network meta−reinforcement learning algorithm based on the original environment of the meta−training phase to set the number of arrays M = 21, the incoming wave angle range [−3°, 3°], Δϕ = 0.1°, λ = 1 m, the array spacing d = λ/2 = 0.5 m, 0 dB SNR, and 40 snapshots to test the two coherent angle estimations and make the error simulation plots of these two angles, selecting 100 test results from 3000 tests, the result of which shows that our proposed method is effective in separating the coherent signals. At the same time, Table 2 gives the detection results of our method based on the three coherent incoming wave angles in the original environment mentioned above; however, the conventional dimensional reduction processing method in Figure 10 can be found not to distinguish the two coherent sources well when testing the last sample of Table 2 in the same test environment, and only one wave peak can be identified.

**Figure 11:**
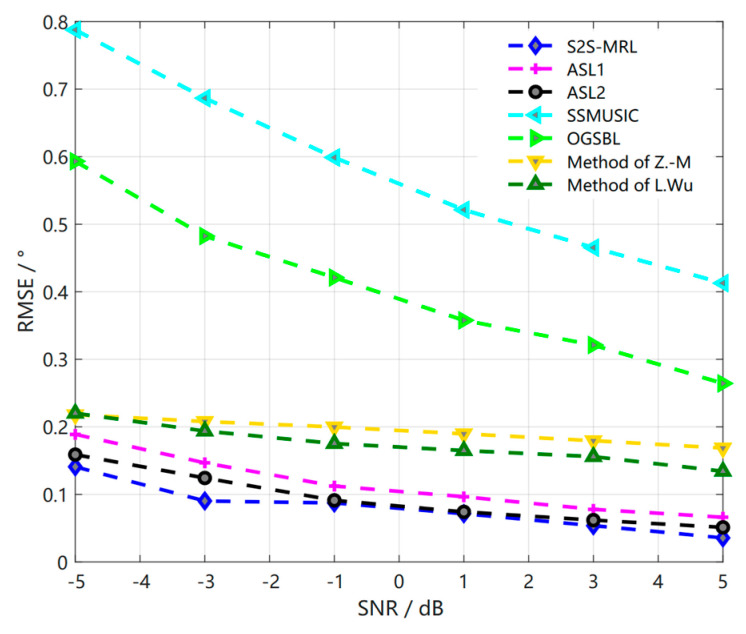
Comparison of RMSE of S2S network meta−reinforcement learning algorithm with other methods in the original environment.

The overall comparison of the RMSE performance of our proposed method with multiple methods in the same parameter environment is performed based on the comparison of the relevant RMSE performance in the original environment of Figure 11 with SNR of −5 dB to 5 dB, where the step size is 2 dB, Δϕ = 0.1°, and 40 snapshots. Among them, the ASLs algorithms in the References [15,16,23], as well as our proposed meta−reinforcement learning algorithm, outperform the physically driven SSMUSIC and OGSBL algorithms, and it is clearly seen that our proposed meta−reinforcement learning algorithm outperforms the other algorithms at low signal-to-noise ratio and its performance remains better than the other algorithms as the signal-to-noise ratio increases. In this process, we can find that the signal-to-noise ratio is close to the ASL2 algorithm at −1 dB and 1 dB, and then the other cases are better than the ASL2 algorithm, which has the best performance among the remaining algorithms.

### 4.3. Performance Testing in New Environments Based on Meta−Testing Phases

**Figure 12:**
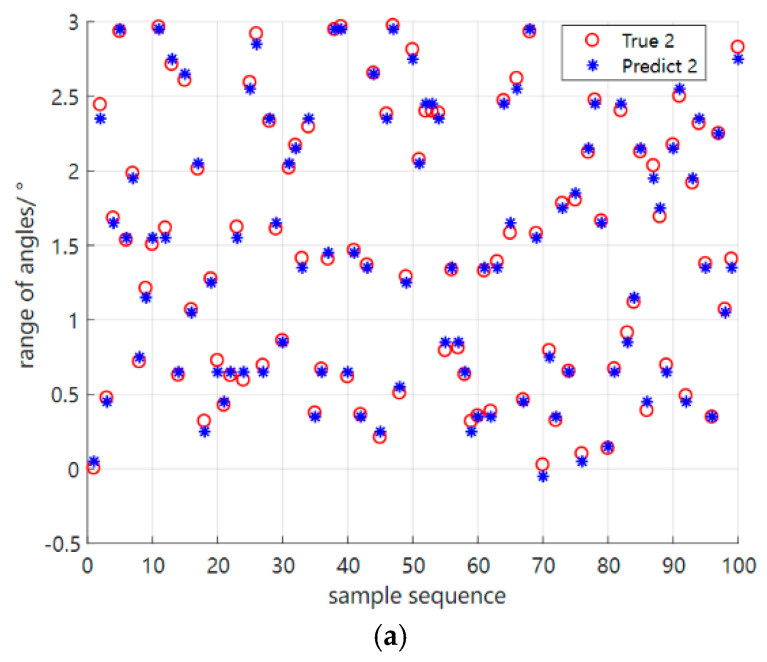

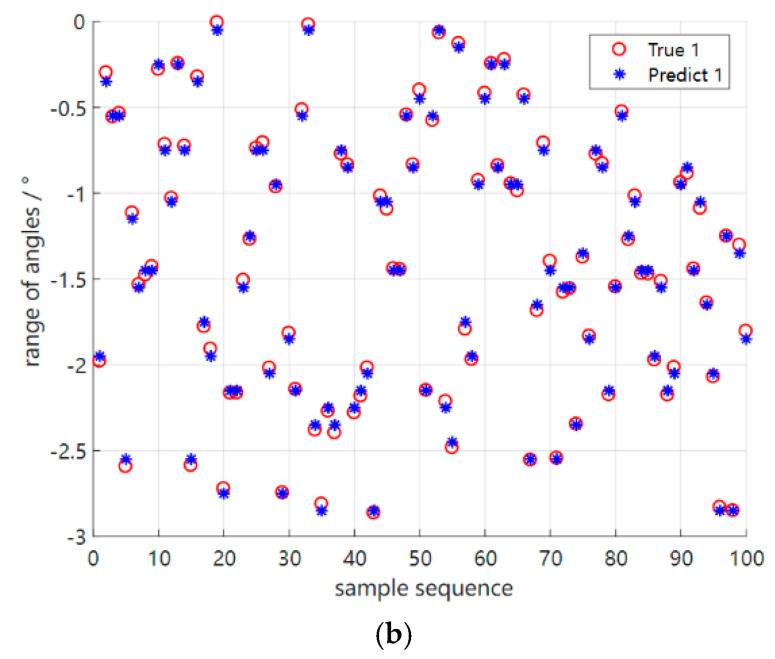
Error plots of the S2S network meta−reinforcement learning algorithm based on two perspectives in the original environment, respectively (**a**,**b**). (**a**) Error plot of the S2S network meta−reinforcement learning algorithm based on the first angle in the new environment. (**b**) Error plot of the S2S network meta−reinforcement learning algorithm based on the second angle in the new environment.

Similarly, in order to demonstrate the generalization capability of the algorithm, the simulation conditions in the new environment based on the meta−testing stage are the same as those in the original environment, except for the SNR of 5 dB. It is also clear from the results in Figure 12 that our proposed methods are effective in separating the coherent signals in the new environment, and we also present in Table 3 the detection results of coherent incoming wave angle for three test data sets based on the new environment.

**Figure 13:**
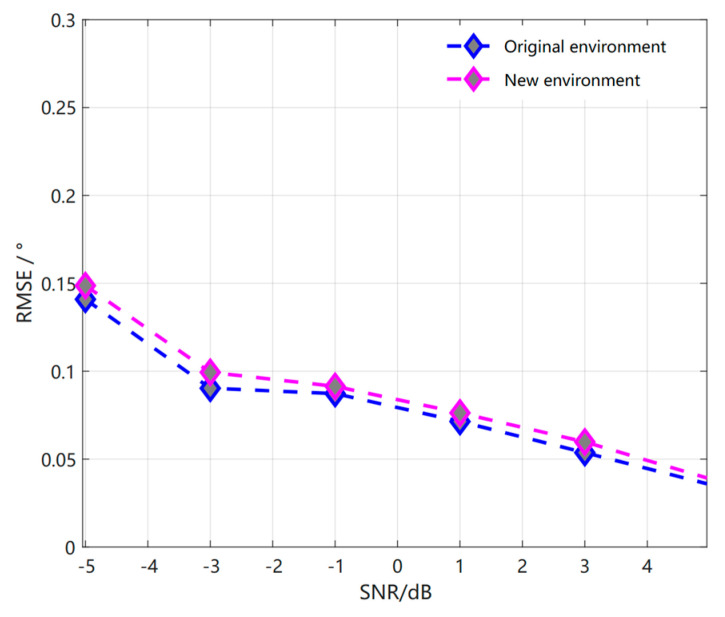
Comparison of RMSE of S2S network meta−reinforcement learning algorithm based on the original environment and the new environment.

Based on the expression λ of the output of the S2S neural network, the two-dimensional peak search of the coherent source is reduced to a one-dimensional search. Table 2 shows the test data taken from the data set under the original environment set SNR of 0 dB in the meta−training phase, and the test data in Table 3 is taken from the new environment set SNR of 5 dB in the meta−test phase with a new data set different from the original environment. Therefore, through the previous analysis and the above table, as well as and the simulation parameters set by the meta−reinforcement learning algorithm in Figure 13 being consistent with Figure 11, as well as being based on the RMSE comparison between the original environment and the new environment, it can be seen that the meta−reinforcement learning algorithm can achieve good results in detection based on the knowledge and experience gained in the meta−training phase when encountering a new environment data set that has never been seen in the meta−testing phase with a small number of training samples and gradient iterations.

### 4.4. Comparison of Test Sample Cost

**Figure 14:**
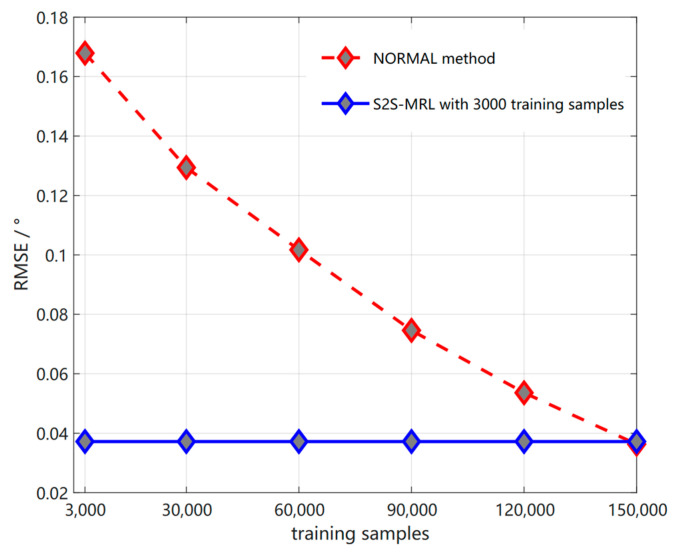
Comparison of RMSE between S2S-MRL and NORMAL method with different training samples and fixed small training samples, respectively.

After that, we will compare the training sample costs of the two methods. The first method is the meta−reinforcement learning algorithm under S2S neural network. The other method is the method of DNN network without meta−reinforcement learning algorithm. The standard for comparing the training sample costs of these two methods is to test a better estimation result and the RMSE value obtained by these two methods is approximate. So as shown in Figure 14, the two methods that need to test two angles in the new environment require different levels of data to achieve a satisfactory accuracy effect. The proposed method only needs to train 150,000 samples in the original environment in the meta−training stage and 3000 samples in the new environment in the meta−testing stage to obtain better estimation results. That is, the S2S neural network meta−reinforcement learning algorithm can quickly adapt to the new environment with a small number of samples and achieve good results in detection, thus obtaining a lower RMSE value. Its value is as shown in the figure 0.0372°. On the contrary, the NORMAL method is initially trained with the same training data set as the S2S neural network meta−reinforcement learning algorithm. We can find that the detection error of the NORMAL method is large and the difference between the RMSE of the algorithm proposed in this paper is large. Obviously, the NORMAL method cannot fully adapt to the data in the new environment with a small number of samples. Therefore, as shown in Figure 14, the NORMAL method, which gradually increases the sample size of the training set in the new environment to 150,000 training sets, is comparable to the RMSE value of the algorithm proposed in this paper, so that the NORMAL method can better adapt to the new environment.

From the above analysis, we can see that when we consider when the signal environment changes, that is, under the new environment, the generalization ability of the NORMAL method, which does not use the meta−reinforcement learning algorithm, is relatively low. Therefore, in this new environment, it is necessary to re-sample signal samples and re-train their models to achieve the RMSE value and its accuracy in Figure 14. On the contrary, the proposed algorithm does not need to retrain the model; that is, it can train samples in the new environment on the basis of prior-existing knowledge. As mentioned before, a good strategy for adapting to new tasks T can be generated by using a small number of samples, a small number of gradient iterations and fine-tuning several gradient steps. Therefore, the two methods differ greatly in the order of sample size used to obtain lower and similar RMSE values. Among them, it can be found from the above Table 4 that the number of training samples of NORMAL method is 50 times that of the algorithm in this paper.

Therefore, when the signal environment changes, the S2S neural network meta−reinforcement learning algorithm will quickly adapt to the new signal environment and give accurate and coherent DOA results due to the aid of meta knowledge. However, the NORMAL method needs a relatively large number of sample data in the new environment to re-train the model to adapt to the new environment; that is, it can give the same accurate coherent DOA results as the S2S neural network meta−reinforcement learning algorithm.

### 4.5. Calculation Complexity and Test Time Analysis

- Calculation complexity

For the S2S-MRL method, once the angle separation is learned, the super-complete spatial spectrum can be calculated. Therefore, the complexity is determined by the following formula (17), which is $O\left(LM^{2}\right)$. For SSMUSIC algorithm, it involves Eivenvaule decomposition operator, and the complexity of SSMUSIC algorithm is about $O\left(M^{3}+LM^{2}\right)$. For OGSBL algorithm, its complexity is related to the number of iterations and convergence conditions. If the number of iterations is *S*, the complexity of OGSBL is about $O\left(S\left(\left(M+1\right)L^{3}+LMT\right)\right)$. Therefore, according to the above and Table 5 below, S2S-MRL method has lower computational complexity than SSMUSIC and OGSBL methods.

B. Test-time analysis

Two coherent signal sources are estimated under different methods of setting the SNR of 5 dB and the number of snapshots of 40. The test-time comparison in Table 6 is made. It can be seen from the table that the S2S-MRL method achieves the highest estimation accuracy and the test time is less than that of the traditional SS-MUSIC algorithm, and slightly less than that of the ASL1 and ASL2 algorithms. This shows that our offline training model can reduce the computational burden without damaging the performance.

## 5. Conclusions

Aiming at the problems of performance degradation or even failure of existing coherent DOA estimation methods based on neural network when training samples are insufficient, the coherent DOA estimation method of small sample S2S neural network based on meta−reinforcement learning is studied. Firstly, the method is based on the coherent angle interval feature vector sequence of the array. According to the characteristics of the angle interval feature vector sequence, it establishes the MDP model and the definition of its state, action, and state transition income, solves the angle interval of the incident signal, and then completes the reduced-dimension spectral peak search, which greatly reduces the computational complexity. At the same time, based on the support of the meta−learning algorithm, the MDP process is converted into a meta−reinforcement learning algorithm, which then solves the problem of small-sample coherent DOA. Secondly, due to the intentional reduction of the quantization error, the sequence length of the required solution increases. According to the simulation results, due to the introduction of the attention mechanism in the S2S neural network expression, the context information between the long sequence tasks can be better utilized, thus contributing to the decoding of coherent sources. The experimental results show that the method based on this paper has the advantages of quickly adapting to the new signal environment, has good robustness to quantization error, and even has great advantages in accuracy and generalization ability in the harsh environment such as low signal-to-noise ratio and small angle domain, so this method is particularly suitable for coherent DOA estimation in challenging application environments.

## Figures and Tables

**Table 1:** Simulation Parameters.

Parameters	Value Description	Parameters	Value Description
Number of m-learning sequences	5	Number of array elements	M = 21
Inner/outer loop learning rate	α/β = 0.002	Snapshots	Snapshots = [100, 80, 60, 40]
Number of training samples	150,000	Number of coherent sources	K = 2
Number of test samples	3000	Beamwidth	5°
Batch size	20	Wave length	λ = 1 m
Gradient descent threshold	5	Interval of Array element	λ/2
MDP return discount factor	γ= 0.9	Incoming wave direction range	[−3°, 3°]
Number of S2S network neurons	units = 256	Quantification unit	Δϕ=[0.05°, 0.1°, 0.15°, 0.2°]
embedding e vector dimension	256	Quantization discrete length	L(Δϕ) = [121, 61, 41, 31]
Over-fitting factor dropout	0.5	Source correlation coefficient	coef =[1,e^(jpi/6),…,e^(j2pi)]
Network hidden unit	Layers = 256	Signal-to-noise ratio 1	SNR=[0 dB, 10 dB, 20 dB]
Encoding layer	Layer1 = 2	Signal-to-noise ratio 2	SNR = [−5 dB, 5 dB] (step = 2 dB)
Decoding layer	Layer2 = 2		

**Table 2:** Identification results of coherent DOA in the original environment.

Original Environment	Direction of Incoming Waves Label (Degree)		Incoming Wave Angle Identification (Degree)	
	Angle of Wave 1	Angle of Wave 2	Angle of Wave 1	Angle of Wave 2
test 1	2.4	−0.2	2.6	−0.2
test 2	2.2	−2.4	2.2	−2.2
test 3	0.2	0.0	0.4	0.0

**Table 3:** Identification results of coherent DOA in the new environment.

New Environment	Direction of Incoming Waves Label (Degree)		Incoming Wave Angle Identification (Degree)	
	Angle of Wave 1	Angle of Wave 2	Angle of Wave 1	Angle of Wave 2
test 1	−2.4	0.8	−2.2	1.1
test 2	−1.6	0.4	−1.6	0.5
test 3	1.4	0.4	1.5	0.4

**Table 4:** Comparison table of training sample size of two methods in approximate RMSE based on the new environment.

Models	RMSE	Training Sample
Normal	0.0363°	150,000
S2S-MRL	0.0372°	3000

**Table 5:** Comparison Table of Calculation Complexity of Different Methods.

Models	Computational Complexity
S2S-MRL	$O\left(LM^{2}\right)$
SSMUSIC	$O\left(M^{3}+LM^{2}\right)$
OGSBL	$O\left(S\left(\left(M+1\right)L^{3}+LMT\right)\right)$

**Table 6:** The test time of two coherent signal sources is estimated at 5 dB SNR.

Models	RMSE	Operation Time(s)
SS-MUSIC	0.4117°	0.0031
ASL1	0.0642°	0.0004
ASL2	0.0513°	0.0004
S2S-MRL	0.0372°	0.0007

## Data Availability

The data presented in this study are available on request from the corresponding author. The data are not publicly available, due to the data in this paper not being from publicly available data sets but obtained from the simulation of the signal models listed in the article.
